# Primary Breast Cancer Tumours Contain High Amounts of IgA1 Immunoglobulin: An Immunohistochemical Analysis of a Possible Carrier of the Tumour-Associated Tn Antigen

**DOI:** 10.1371/journal.pone.0061749

**Published:** 2013-04-18

**Authors:** Charlotte Welinder, Bo Baldetorp, Ola Blixt, Dorthe Grabau, Bo Jansson

**Affiliations:** 1 Department of Oncology, Clinical Sciences, Lund University, Lund, Sweden; 2 Copenhagen Center for Glycomics, Department of Cellular & Molecular Medicine, University of Copenhagen, Copenhagen, Denmark; 3 Department of Pathology, Skåne University Hospital, Lund, Sweden; INRS, Canada

## Abstract

The Tn antigen (GalNAc alpha-O-Ser/Thr) as defined by the binding of the lectin, helix pomatia agglutinin (HPA) or anti-Tn monoclonal antibodies, is known to be exposed in a majority of cancers, and it has also been shown to correlate positively with the metastatic capacity in breast carcinoma. The short O-glycan that forms the antigen is carried by a number of different proteins. One potential carrier of the Tn antigen is immunoglobulin A1 (IgA1), which we surprisingly found in tumour cells of the invasive parts of primary breast carcinoma. Conventional immunohistochemical analysis of paraffin-embedded sections from primary breast cancers showed IgA1 to be present in the cytoplasm and plasma membrane of 35 out of 36 individual primary tumours. The immunohistochemical staining of HPA and anti-Tn antibody (GOD3-2C4) did to some extent overlap with the presence of IgA1 in the tumours, but differences were seen in the percentage of stained cells and in the staining pattern in the different breast cancers analysed. Anti-Tn antibody and HPA were also shown to specifically bind to a number of possible constellations of the Tn antigen in the hinge region of IgA1. Both reagents could also detect the presence of Tn positive IgA in serum. On average 51% of the tumour cells in the individual breast cancer tumour sections showed staining for IgA1. The overall amount of staining in the invasive part of the tumour with the anti Tn antibody was 67%, and 93% with HPA. The intra-expression or uptake of IgA1 in breast cancer makes it a new potential carrier of the tumour associated and immunogenic Tn antigen.

## Introduction

The Tn antigen CD175 is generally defined as (GalNAc alpha-O-Ser/Thr) or as a cluster of the same glycan. Tn antigen is the result of an abnormal O-glycosylation. Tumour-associated changes such as the Tn antigen and other changes in O-glycosylation have been found to be immunogenic and present on a variety of proteins, e.g. CD43 in T-cell leukaemia cells [1], MUC-1 in colon cancer [2], CD44 in breast carcinoma [3] and nucleolin in melanoma [4]. The majority of all carcinomas, 80–90%, are positive for the Tn antigen as defined by the lectin HPA. Furthermore, up-regulation of the Tn antigen in tumours is associated with poor prognosis [3], [5], [6], [7]. Previously HPA affinity chromatography of a number of solubilised breast cancer tumours followed by SDS-PAGE and peptide sequencing have identified a major Tn-carrying 55 kDa protein in breast cancer metastatic tissue lysate as the heavy chain of IgA1 [8]. The O-glycosylation in IgA1is normally found in the hinge region of immunoglobulin, which may theoretically carry a maximum of nine O-glycosylations and it makes IgA1 a potential carrier of Tn antigen and potential target for an anti-tumour response [9]. The therapeutic usefulness of an anti-Tn antibody in passive immunotherapy has been illustrated *in vivo* with different animal models. Treatment with the anti Tn antibody GOD3-2C4 of SCID mice grafted with a human tumour cell line significantly reduced the growth rate of the tumor and when combined with cyclophosphamide another chimeric anti Tn antibody induced complete rejection of a murine mammary tumor in immune competent animals [10], [11].

We have performed a short study that demonstrates high frequency of IgA1 positive cells in primary breast tumours. IgA1 was found to be present in both the cytoplasm and plasma membrane of 35 out of 36 individual breast cancer tumours The percentage and intensity of staining correlated to some extent with the staining intensity patterns of HPA and GOD-2C4 indicating, as expected, that IgA1 is not the only protein that carries the Tn antigen in the tumour. We also demonstrate in this study that HPA and anti Tn antibody GOD3-2C4 bind different glycoforms of the GalNAc alpha-O-Ser/Thr in the hinge region of IgA.

## Materials and Methods

### Reagents and cell lines

The monoclonal M4D8 anti-human IgA1 [12] was obtained from Margaret Goodall at The Division of Immunity & Infection University of Birmingham B15 2TT United Kingdom ., the anti-human poly-Ig receptor- (pIgR] biotinylated antibody BAF2717, from R&D Systems Europe Ltd (Abingdon, United Kingdom), and the negative control mouse IgG from Jacksson ImmunoResearch Europe Ltd (Suffolk, United Kingdom) . The anti-Tn monoclonal antibody GOD3-2C4 was produced in-house [10]. The biotinylated lectin, HPA, was purchased from EY Laboratories, Inc. (San Mateo, CA, USA). T47D and MCF-7 breast carcinoma cell lines were obtained from the American Type Culture Collection (ATCC).

### Immunohistochemistry

Briefly, the tissue sections (4 µm) were de-paraffinized in xylene and rehydrated stepwise in ethanol and distilled water. Before staining, the sections were treated with antigen retrieval buffer (S1699, Dako, Glostrup, Denmark) in a 2100-Retriver (PickCell Laboratories, HistoLab, Västra Frölunda, Sweden). The slides were then allowed to cool for at least 20 min. An automated immunostainer (TechMate 500Plus, Dako) was used for the staining procedure: 30 minutes' staining for the primary antibody M4D8 anti-human IgA1 (dilution 1∶2000), the anti-human pIgR-biotinylated antibody (dilution 10 µg/mL), the negative control mouse IgG (dilution 10 µg/mL), GOD3-2C4 (dilution 10 µg/mL), biotinylated HPA (dilution 25 µg/mL) and the secondary antibody. Staining was visualized with the EnVision TM Detection system (K5001 for the biotinylated antibodies and K5007 for the other antibodies, Dako, Denmark). The slides were counterstained with haematoxylin.

The percentage of invasive tumour cells stained in each slide was evaluated on a continuous scale (0–100%). Staining intensity was assessed semi-quantitatively: 0 = completely negative slide, 1 = weak, 2 = moderate and 3 = strong intensity. Magnifications ranging from 4 to 40 times were used during scoring. The histological grade was assessed according to Elston et al. [13]. The majority of the tumours were invasive ductal breast cancers. Sample 6 was classified as mucinous and sample 35 as tubular cancer. Samples 2 and 35 were recidiv, while the remainder of the tumours were primary lesions.

### Microarray analysis

The fine specificity of GOD3-2C4 and HPA for different Tn antigens was analysed using a glycopeptide array. The assay was performed as described previously [14], with a synthetic screening microarray platform with O-glycosylated 20-amino-acid sequences from the hinge region of the IgA. Briefly, the construction of the array is based on chemical solid-phase glycopeptide synthesis and selective the enrichment of defined glycopeptide on a hydrogel-coated microarray glass slide. Each slide contains the glycopeptides given in Table 1. Peptide no.1 is the non-glycosylated peptide representing the background binding. Each slide was incubated for one hour with 10 µg/mL GOD3-2C4 or HPA in PBS, pH 7.4. The GOD3-2C4 antibody was detected with Cy3-conjugated goat anti-mouse IgG (H+L) (Jackson ImmunoResearch Laboratories, Inc., (Suffolk, United Kingdom) diluted 1∶1000. The biotinylated HPA was detected with streptavidin-Alexa Fluor 488 (Invitrogen, Carlsbad, CA, USA; diluted 1∶5000). All incubation steps were separated by two washing steps in PBS containing 0.05% Tween-20 and one washing step in PBS. After the final washing step, the slides were rinsed in water and air dried. Finally, the slides were scanned (Pro Scan Array HT Microarray, Perkin Elmer Life and Analytical Sciences, MA, USA) and analysed using image analysis software (Scan Array Express, v 3.0, Perkin Elmer Life and Analytical Sciences).

**Table 1:** The 44 different IgA hinge glycopeptides tested with Helix Pomatia Lectin and anti-Tn antibody.

No.	IgA hinge glycopeptide
1	VPSTPPTPSPSTPPTPSPSA
2	VP**S**TPPTPSPSTPPTPSPSA
3	VPS**T**PPTPSPSTPPTPSPSA
4	VPSTPP**T**PSPSTPPTPSPSA
5	VPSTPPTP**S**PSTPPTPSPSA
6	VPSTPPTPSP**S**TPPTPSPSA
7	VPSTPPTPSPS**T**PPTPSPSA
8	VPSTPPTPSPSTPP**T**PSPSA
9	VPSTPPTPSPSTPPTP**S**PSA
10	VPSTPPTPSPSTPPTPSP**S**A
11	VP**S**TPPTPSP**S**TPPTPSPSA
12	VP**S**TPPTPSPSTPP**T**PSPSA
13	VPS**T**PPTPSPSTPP**T**PSPSA
14	VPSTPP**T**PSP**S**TPPTPSPSA
15	VPSTPPTP**S**P**S**TPPTPSPSA
16	VPSTPPTPSP**ST**PPTPSPSA
17	VPSTPPTPSPS**T**PP**T**PSPSA
18	VPSTPPTP**S**PSTPP**T**PSPSA
19	VPSTPPTPSP**S**TPP**T**PSPSA
20	VPS**T**PPTPSP**S**TPPTPSPSA
21	VPSTPPTP**S**P**S**TPPTPSPSA
22	VPSTPP**T**PSPSTPP**T**PSPSA
23	VP**ST**PPTPSP**S**TPPTPSPSA
24	VP**ST**PPTPSPSTPP**T**PSPSA
25	VPS**T**PP**T**PSP**S**TPPTPSPSA
26	VPS**T**PP**T**PSPSTPP**T**PSPSA
27	VPSTPP**T**P**S**PSTPP**T**PSPSA
28	VPSTPPTPSP**ST**PP**T**PSPSA
29	VPSTPPTP**S**P**ST**PP**T**PSPSA
30	VPSTPP**T**P**S**P**ST**PPTPSPSA
31	VPS**T**PP**T**P**S**P**ST**PPTPSPSA
32	VPS**T**PP**T**P**S**PS**T**PPTPSPSA
33	VP**ST**PP**T**PSP**S**TPPTPSPSA
34	VP**ST**PP**T**PSPSTPP**T**PSPSA
35	VPS**T**PP**T**P**S**P**S**TPPTPSPSA
36	VPS**T**PP**T**P**S**PSTPP**T**PSPSA
37	VPSTPP**T**P**S**P**S**TPP**T**PSPSA
38	VP**ST**PP**T**P**S**P**S**TPPTPSPSA
39	VP**ST**PP**T**P**S**PSTPP**T**PSPSA
40	VPS**T**PP**T**P**S**P**S**TPP**T**PSPSA
41	VPSTPP**T**P**S**P**ST**PP**T**PSPSA
42	VP**ST**PP**T**P**S**P**ST**PPTPSPSA
43	VP**ST**PP**T**P**S**P**S**TPP**T**PSPSA
44	VP**ST**PP**T**P**S**P**ST**PP**T**PSPSA
45	VPS**T**PP**T**P**S**P**ST**PP**T**PSPSA
46	VP**ST**PP**T**P**S**P**S**TPP**T**PSPSA

Bold and S and T indicate O-glycosylation with Ga1NAc.

### Cell culturing

The human carcinoma cell lines MCF-7 and T47D were purchased from the ATCC (Rockville, MD, USA) and cultivated at 37°C in RPMI 1640 supplemented with 10% FCS.

### ELISA

Binding to IgA was tested with a sandwich ELISA using HPA, GOD3-2C4 or polyclonal anti-IgA as a catcher antibody. White 96-well test plates (Lumitrac 600, Greiner-Bio One, Frickenhausen, Germany) were coated with 50 µL of 2 µg/mL antibody or lectin in 0.1 M sodium carbonate buffer, pH 9 at +4°C overnight. All following washing steps and dilution of reagents were performed with PBS containing 0.05% Tween 20. The plates were washed and then incubated for one hour with 50 µL of diluted (1∶5) conditioned culture supernatants, medium or serum. After washing, bound IgA1 antibody was detected with HRP-conjugated rabbit anti-IgA (P-0216; DAKO). After the final washing step the bound antibody was visualized with SuperSignal Femto Maximum substrate for chemiluminescence for ELISA detection (Thermo Scientific, USA). The plates were read on a Wallac Victor II Fluorescence plate reader (PerkinElmer, USA).

### Ethics statement

This study was approved by the Ethics Committee of Lund University (LU 240-01).

## Results

Paraffin sections from 36 different breast cancer tumours were stained for IgA1, Tn antigen, pIgR and HPA. A summary of the results is presented in Table 2. The percentage and intensity of positive staining of tumour cells in the invasive part of each tissue section was analysed. Immunostaining with M4D8 (for IgA1), HPA, pIgR and GOD3-2C4 (for Tn) is compared with non-binding mouse Ig. The percentage of stained invasive tumour cells was evaluated on a continuous scale (0–100%), while the relative intensity of tissue staining was classified as 0–3.

**Table 2:** Summary of Histological Immunohistochemical Results.

Sample	Hormone receptor	Histology grade	IgA1, %	Int.	pIgR, %	Int.	HPA, %	Int.	Tn, %	Int.
1	−	2	40	2	40	2	100	1	80	2
2	+	3	40	3	40	3	100	2	60	2
3	+	3	30	1	1	1	100	2	90	3
4	+	1	80	3	60	1	100	2	100	2
5	+	2	60	3	0	0	50	3	30	3
6	+	1	5	2	0	0	100	2	0	0
7	−	2	80	3	20	2	100	1	10	1
8	+	3	50	3	80	3	100	3	70	3
9	+	3	20	3	10	3	90	2	60	2
10	+	1	20	3	70	3	90	1	20	2
11	−	1	30	2	10	3	100	1	10	3
12	+	3	2	1	5	3	100	3	70	2
13	+	2	70	2	60	2	100	3	90	2
14	+	3	80	3	0	0	80	1	70	1
15	+	1	90	3	50	2	100	3	100	2
16	+	1	50	2	20	1	100	2	90	2
17	+	1	30	3	1	1	90	2	30	3
18	+	2	50	2	40	1	50	1	30	2
19	+	2	10	1	30	3	100	3	80	3
20	+	3	40	2	1	1	100	3	100	3
21	−	3	1	2	0	0	60	2	20	2
22	+	1	50	2	50	2	100	3	90	3
23	+	2	80	3	60	1	100	3	100	3
24	+	1	60	2	30	2	100	2	100	2
25	+	2	80	2	10	1	100	2	30	2
26	+	1	50	1	5	1	80	1	90	1
27	+	3	10	1	20	2	100	3	70	3
28	−	2	100	3	5	1	100	3	100	3
29	−	1	70	2	60	1	100	1	50	2
30	−	3	95	2	20	2	100	3	90	2
31	+	2	60	2	80	1	100	2	100	2
32	+	3	90	2	10	1	90	1	1	1
33	+	2	0	0	0	0	100	2	100	1
34	0	3	90	2	30	3	100	2	100	2
35	+	1	50	2	20	3	80	1	90	1
36	+	3	70	3	40	2	100	3	90	3

Intensity = Int., Hormone receptor = Oestrogen and progesterone receptors.

**Figure 1:**
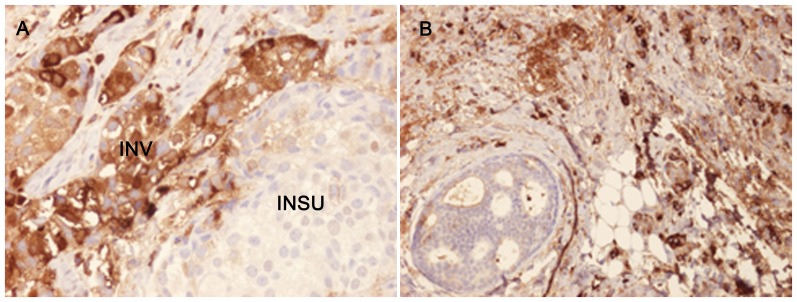
Immunohistochemical staining of breast tumour specimens showing the presence of IgA1 in the invasive part of the tumour. A) In Sample 2, 40% of the tumour cells in the invasive part were stained with anti-IgA1 with a relative intensity of 3. Strong cytoplasmic and plasma membrane staining of IgA1 is observed in the invasive part of the section (INV) but only very weak staining in the *in situ* part (INSU). B) In Sample 7, 80% of the cells were IgA1-positive with a relative intensity of 3.

**Figure 2:**
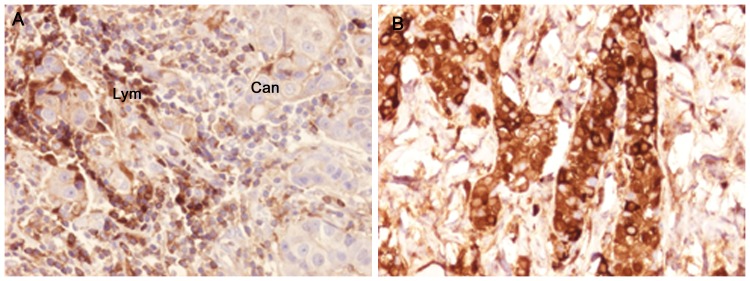
Immunohistochemical staining of specimens from individual breast cancer tumours showing the presence of IgA1 in both lymphocytes and tumour cells. A) A section showing weak positive staining of cancer cells (Can) and intensively stained lymphocytes (lym). B) Intense anti- IgA1 staining of cancer cells in Sample 28, an ER/PGR-negative tumour in which 100% of the invasive part of the section was regarded as being maximally stained for IgA1 with an intensity of 3.

**Figure 3:**
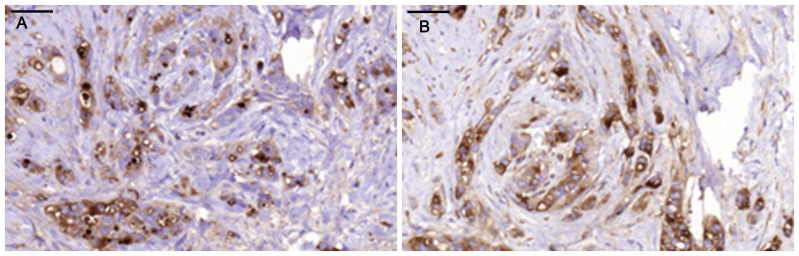
Intermediate staining percentage of IgA1-positive tumour cells in Sample 1 compared to staining for HPA. **Scale bar = 50 µm** A) Anti-IgA1, B) HPA.

**Figure 4:**
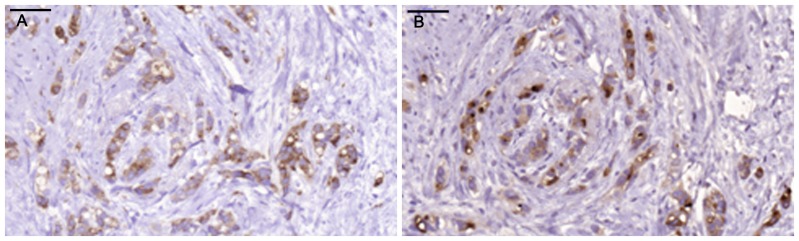
Staining with anti-Tn and anti-pIgR in Sample 1. **Scale bar = 50 µm.** A) Anti-Tn, B) Anti-pIgR.

**Figure 5:**
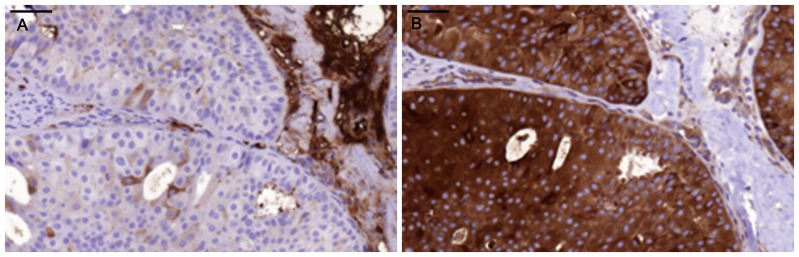
Very low percentage of IgA1-positive tumour cells in tumour Sample 33. **Scale bar = 50 µm.** A) Anti.IgA1, B) HPA.

**Figure 6:**
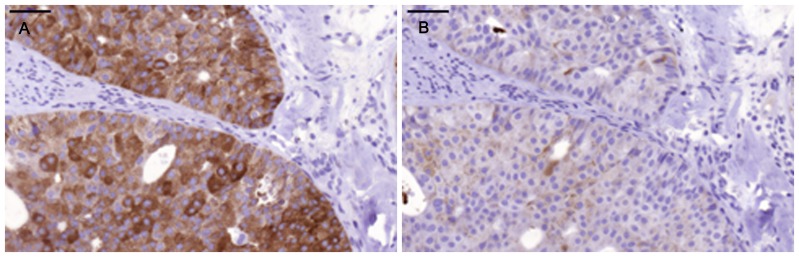
Staining with anti-Tn and anti-pIgR in Sample 33. **Scale bar = 50 µm.** A) Anti-Tn, B) Anti-pIgR.

**Figure 7:**
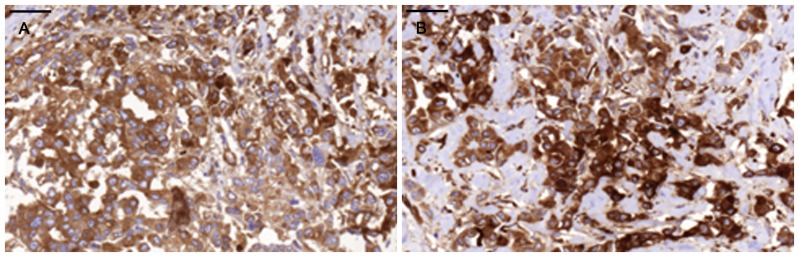
High percentage of IgA1-positive tumour cells compared with staining with HPA in tumour Sample 30. **Scale bar = 50 µm.** A) Anti-IgA1, B) HPA.

**Figure 8:**
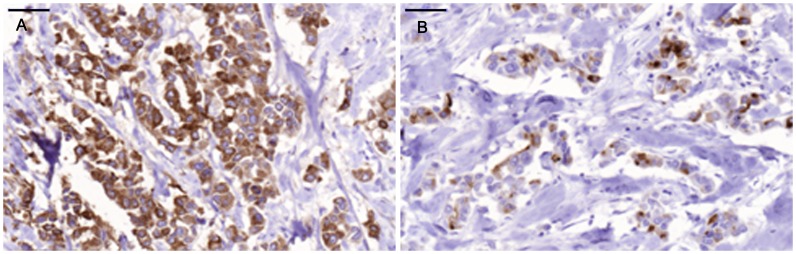
Staining with anti-Tn and anti-pIgR in Sample 30. **Scale bar = 50 µm** A) Anti-Tn, B) Anti-pIgR.

Positive staining for IgA1 was seen in the majority of the breast cancer sections. Sections morphologically classified as invasive were more intensely stained than other parts classified as cancer *in situ* (Figure 1A). Figures 1 and 2 illustrates four different breast cancer tumour samples stained with anti-IgA1, showing different intensity and amounts of stained cancer cells in the invasive part of the tumour. The percentage of IgA1-positive cells ranged from 0–100%, with an overall average of 51%. Binding was frequently seen in both the cytoplasm and plasma membrane of the breast cancer tumour cells, as can be seen in Figures 1 and 2. Breast cancer cells in the invasive part also stained positive for binding of HPA and GOD3-2C4, to varying degrees. All three reagents, anti IgA1, HPA and GOD3-2C4, showed overlapping staining of breast cancer tumour cells, but with clear differences in intensity, proportion and inter-cellular distribution between different breast cancer tumour sections. The sections also stained positive for pIgR, one of the receptors for IgA. The majority of the invasive tumour sections stained positive for both IgA1 and pIgR, but there was no obvious correlation between the frequency of expression. Two tumours stained negative for pIgR but still stained intensively for IgA1 (Samples 5 and 14 in Table 2). Examples of staining patterns for IgA1, HPA, GOD3-2C4 and pIgR are shown for three different breast cancer tumour samples in Figures 3, 4, 5, 6, 7, 8. The percentage staining for IgA1 was 40% in the invasive part of the tumour in Sample 1, 0% in Sample 33 and 95% in Sample 30 (Figures 3A, 5A and 7A). Forty percent of the cells were stained for IgA1 and pIgR in tumour Sample 1 (Figures 3A and 4B). The IgA1-negative tumour (Sample 33) showed no staining for pIgR (Figures 5A and 6B). Ninety-five percent of Sample 30 was stained for IgA1, but much less for pIgR, being only 20% (Figures 7A and 8B). HPA and GOD3-2C4 staining seems to be similar to each other in the three different breast cancer tumour samples shown in Figures 3B, 4A, 5B, 6A, 7B and 8A. However, this was not the case for all breast cancer tumour samples analysed (see Table 2). A majority of the invasive tumour cells showed high intensity staining with HPA, while less intensity and frequency was seen with the monoclonal GOD3-2C4 antibody.

**Figure 9:**
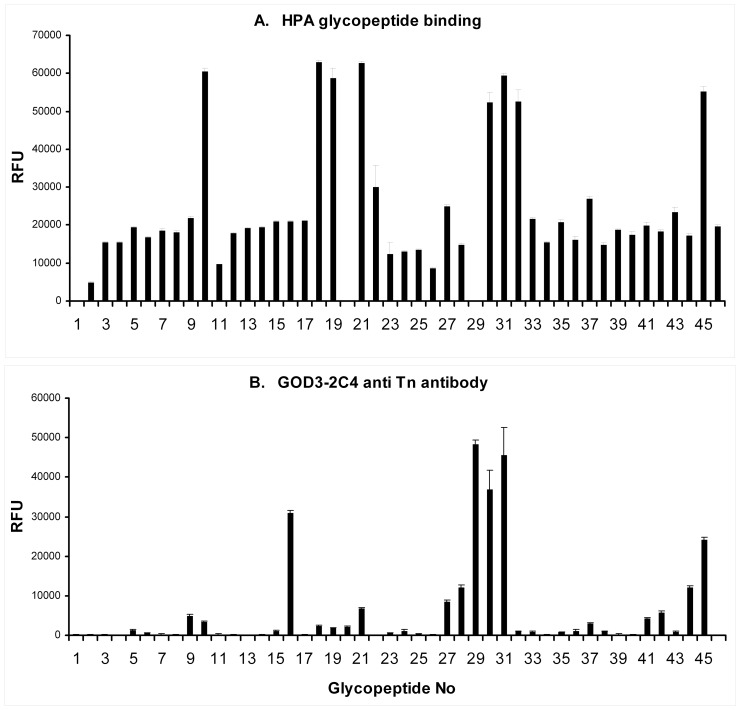
Helix Pomatia Lectin and GOD3-2C4 (anti-Tn antibody) glycopeptide array. Relative fluorescence signal (RFU). The mean value of five individual measurements is given for each glycopeptide. The numbers of each specific Tn peptide are listed in Table 1. A) HPA, B) GOD3-2C4.

**Figure 10:**
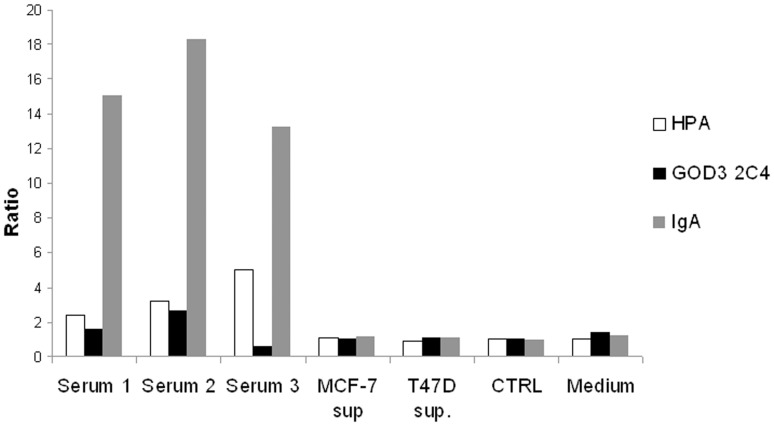
Results of sandwich IgA ELISA of human serum and cultivation supernatant from dense cultures of MCF-7 and T47D breast carcinoma cell lines. Serum or conditioned culture supernatant from two different breast carcinoma cell lines (MCF-7 sup. and T47D sup.) were incubated in test plates coated with HPA, GOD3-2C4 (Tn) or polyclonal anti-IgA antibody (IGA). A polyclonal anti-IgA HRP conjugated antibody was used for detection, and the results are presented as the quotient between a coated non-binding control and the relevant catcher reagent. Mouse serum (CTRL) and fresh RPMI 1640 cultivation medium (Medium) were used as negative controls.

The anti-Tn antibody, GOD3-2C4, and HPA were also tested for specific binding to 46 different glycoforms of the Tn antigen expressed on a microarray platform. Various O-glycoforms of the 20-amino-acid IgA1 heavy-chain hinge region peptide, VPSTPPTPSPSTPPTPSPSA were tested. GOD3-2C4 shows very selective binding to peptides expressing two or more adjacent GalNAc alpha-O-Ser/Thr carbohydrate epitopes while HPA has a much broader binding pattern allowing binding to single GalNAc alpha-O-Ser/Thr carbohydrate epitopes (Table 1 and Figure 9). Using different sandwich ELISAs, we were also able to show that some healthy blood donors have Tn-positive IgA in the circulation. However, no IgA was detected in culture supernatant from two different breast cancer cell lines (Figure 10).

## Discussion

Tn antigen expression is correlated with poor prognosis regarding the metastatic potential of breast cancer [15]. CD44 and MUC-1 are already known to be Tn-positive proteins in breast cancer [3]. These proteins play a role in adhesion and/or migration, and it has been suggested that changes in their O-glycosylation might influence the function and mobility of cancer cells [16]. Streets et al. [8] found a dominating 55-kDa band in SDS-PAGE analysis after HPA affinity chromatography of lysate from metastatic breast cancer tissue. The 55-kDa band was identified as the heavy chain of IgA1. They also reported that IgA1 extracted from normal control tissues bound much less to HPA. Increased concentrations in serum of human IgG, IgM and IgA have been reported in patients with epithelial carcinomas [17], and also the occasional intra-cellular presence of secretory component and IgA in breast carcinoma [18] but no conclusive data has been presented. However, when applying a conventional immunohistochemical technique we found very high amounts of IgA1 in the majority of breast cancer tissues examined. Thirty five individual samples out of thirty six tested were positive. The breast cancer tumour cells in the invasive parts of the tumour were more frequently IgA1-positive than those in the *in situ* parts of the tumour (Figure 1A). Based on our observations, IgA1 expression or uptake in invasive primary breast cancer cells seems to be a frequent phenomenon. The presence of IgA in tumour sections was confirmed in a small number of tissue sections with a polyclonal anti human IgA reagents (data not shown) There may be different explanations of the enrichment of IgA1 in tumour cells, such as specific binding of the antibody to tumour cells [19] or the active uptake of IgA1. The biological functions of immunoglobulin IgA1 antibodies depend primarily on their interaction with cell surface receptors, and several cancer cell receptors are available for binding and internalization of IgA1. Fc αRI (CD89), poly-IgR, Fc α/μR, asialo-glycoprotein receptor and the transferrin receptor [20] all have the capacity to transfer IgA1 into the cell. For some of the breast cancer tumours studied here, a correlation was seen between the staining intensity (Figures 3A and 4B), or lack of staining (Figures 5A and 6B), between pIgR and IgA1 in the cancer cells. Some of the breast cancer tumours stained intensively for IgA1 but much weaker for pIgR (an example of such a tumour is shown in Figures 7A and 8B). This could indicate there is at least two different receptors involved or that the pIgR is down regulated.

A third explanation for the uptake of IgA1 may be the reported capacity of epithelial cancer cells to express endogenous immunoglobulin [21]. There are a number of publications on the potential of cancer cell lines to produce immunoglobulin and other B-cell-associated proteins [21]. According to one study, cancer cell lines have the capacity to express heavy-chain IgA1 [22]. In previous work using the highly sensitive RT-nested PCR method it was shown that some cancer cell lines transcribe both immunoglobulin and T-cell receptor genes [23]. Since then, there have been reports of several cancer cell lines that express immunoglobulin alpha chains, both in the cytoplasm and in secreted form in the cultured supernatants of cancer cell lines [22]. Transcription of the immunoglobulin A1 heavy chain (SNC73), together with the light κ and λ chains, has also been detected with RT-nested PCR and immunohistochemistry in human epithelium-derived tumour cells, including the breast carcinoma cell line MCF-7 [24]. Using a variety of techniques such as immunohistochemical analysis, *in situ* hybridization and laser capture micro-dissection, Qiu et al. have demonstrated that established epithelial cancer lines including breast cancer can produce IgG in both cytoplasmic and secreted forms [25]. However, some of these results are in contrast to observations made by other researchers. When epithelial cancer cells were analysed after being sorted with FACS as EpCAM + cells from cultured cancer cell lines they were found positive for Ig mRNA but no Ig protein expression could be detected in flow cytometry, indicating very low protein expression of IgA [26]. We have obtained similar results when no IgA could be detected in any of the fixed permeabilized tumour cell line tested using flow cytometry (data not shown). We could neither detect any IgA protein in the supernatants from the cultured MCF-7 or T47D cell lines (Figure 10), as has been claimed previously [27]. Perhaps are cultivation conditions and the use of very specific sub clones of cancer cells lines very critical for the production of an efficient amounts of IgA1 *in vitro* and the most optimal conditions are only met in some parts of the tumour “in vivo”. Alternatively the major part of the IgA1 seen in the tumour cells originates from the tumour uptake of surrounding proteins.

Human IgA1 contains both N- and O-glycosylation sites and carries nine potential O-glycosylation sites in its heavy-chain hinge region, but only a maximum of five sites are believed to be glycosylated [5], [9], [28]. Each attached O-glycan has a core of GalNAc alpha-O-Ser/Thr (Tn antigen) typically linked to galactose and one or two sialic acid residues shielding the Tn epitope. Abnormally glycosylated IgA1, e.g. Tn-positive IgA1, is known to play a part in autoimmune diseases such as IgA nephropathy [29]. In patients with IgA nephropathy abnormalities in O-glycan biosynthesis result in exposure of the immunogenic Tn antigen by auto-antibodies, resulting in immune complex formation and deposition in the kidneys, leading to kidney failure [29] and it is tempting to think the expression of aberrant O- glycosylation on IgA1 in a similar way could constitute the target of an anti-tumour immune response [30].

It has also been suggested by that the presence of immunoglobulins could be advantageous for a tumour cell. Li et al. [27] reported that transfecting MCF-7 cells with small interfering RNA (siRNA) blocking the production of Ig inhibited their growth, and that the presence of cancerous Ig specifically reduced antibody-dependent cell-mediated cytotoxicity induced by an anti-human EGF receptor antibody in a dose-dependent manner, suggesting that tumour-associated Ig has a protective role. Blocking of the cancer derived IgA have been shown to suppress growth and viability of cancer cells [22]. Furthermore, the blocking of this tumour-derived IgG increased programmed cell death and inhibited tumour growth *in vitro* and in xeno-transplants *in vivo* [25].The observation that breast cancer cells contain high amounts of IgA1 *in vivo* needs further investigations of the origin, clonality and significance of this tumour associated immunoglobulin. As IgA1 is a potential carrier of the Tn antigen it may provide a target or a blocking decoy for antibody-based therapy.

GOD3-2C4 is monoclonal antibody specific to the Tn antigen [10] and it preferentially binds adjacent GalNAc alpha-O-Ser/Thr epitopes in the hinge region of IgA1, but its binding pattern in the array also indicates a preference for some amino acid sequences, indicated by its specificity for inner cluster (glycopeptides 16, 28, 29, 30, 31, 41, 42, 44, 45) but not flanking regions (glycopeptide 23, 24 and 34), (Figure 9B).

GOD3-2C4 also binds different known Tn-positive proteins from different cancer cell lines, e.g. CD44 and mucins (data not shown). The difference between the reagents is also seen in the immunohistochemistry staining patterns of HPA and GOD3-2C4 which did not always overlap in the breast cancer samples (Table 2). This could be explained by the difference in fine specificity of the two reagents (Figure 9), but also because HPA is known to bind blood group A, while GOD3-2C4 is not cross reactive [10].

Both HPA and GOD3-2C4 bind the Tn antigen on the IgA1 hinge region and in the case of GOD3-2C4 it is clear that clustered bis-GalNAc structure are preferred. HPA has of course a broader reaction pattern. The observation that glycopeptide 20 and 29 are negative might be experimental artifact and has to be examined further. Although these peptides are glycosylated, it might very well be that the glycan structure is sterically hindered by an un-favourable conformation. A recent publication noticed a similar situation for HPA microarray experiment to IgA hinge glycopeptide (Fig. 4 in reference [31]). Both reagents also recognize a portion of the circulating IgA1 proteins in healthy blood donors.

The anti-Tn antibody has *in vitro* and *in vivo* effects on the growth of tumours, and GOD3-2C4 was the first anti-Tn antibody to show an *in vivo* reduction of growth of a xeno-transplanted solid tumour [10]. A more dramatic and convincing therapeutic effect was seen with the Tn-antigen-specific chimeric monoclonal antibody (Chi-Tn, originally denoted 83D4) in a syngeneic breast cancer tumour when combined with cyclophosphamide [11].

## Conclusions

The cytosol and plasma membrane of invasive breast cancer cells frequently contain IgA1, a carrier of the immunogenic Tn antigen. The origin and possible function of the observed tumour-associated IgA1 are unknown, but its relatively high abundance makes it an interesting biomarker and potential therapeutic target
